# Evaluating the Role of Neurogenic Locus Notch Homolog Protein 1 in Oral Cancer Progression and Therapeutic Opportunities

**DOI:** 10.7759/cureus.86149

**Published:** 2025-06-16

**Authors:** Mostafa Ahmed Abdellah Ahmed, Tayyaba Rafiq, Noor Ul Ain Rashid, Madeeha Minhas, Seemi Tanvir, Sajid Ali Majeedano, Asma Ali, Muhmmad Hussain Shah

**Affiliations:** 1 General Surgery, Frimley Health NHS Foundation Trust, Frimley, GBR; 2 Oral and Maxillofacial Surgery, Lahore Medical and Dental College, Lahore, PAK; 3 General Surgery, Akhtar Saeed Trust Hospital, Lahore, PAK; 4 College of Science and Health Professions, King Saud Bin Abdulaziz University for Health Sciences, Jeddah, SAU; 5 Pathology, King Abdullah International Medical Research Center, Jeddah, SAU; 6 Pathology, Ministry of National Guard Health Affairs, Jeddah, SAU; 7 Pathology, Margalla Institute of Health Sciences, Rawalpindi, PAK; 8 Oral Medicine, Muhammad Medical and Dental College, Mirpurkhas, PAK; 9 Pathology, School of Biochemistry and Biotechnology, University of the Punjab, Lahore, PAK; 10 Pathology, Dow University of Health Sciences, Dow International Medical College, Karachi, PAK

**Keywords:** immunity, mucoepidermoid carcinoma, notch1, oral carcinoma, pathways, regulatory, squamous cell

## Abstract

Background and aim: The most common type of malignancy on a global scale is oral cancer, as its incidence level is high worldwide. The neurogenic locus notch homolog protein 1 (NOTCH1) plays a key role in cell maturation and the regulation of the immune system; however, its functions are still not fully understood. This research evaluated the expression of NOTCH1 across two types of oral cancer (oral squamous cell carcinoma {OSCC} versus mucoepidermoid carcinoma {MEC}) and healthy controls to observe its correlation with immune suppression and patterns of disease progression.

Methods: This case-control study was conducted over a seven-month period from March 2023 to September 2023. A total of 120 participants were enrolled and divided into three equal groups, i.e., 40/120 (33.34%) OSCC cases, 40/120 (33.33%) MEC cases, and 40/120 (33.33%) healthy controls. The RNA extraction was done using a protocol, and blood and tumor tissues were taken for the purpose, after which cDNA was produced. The analysis was performed using NOTCH1-specific primers by quantitative real-time PCR (RT-qPCR). SPSS version 20 (Armonk, NY: IBM Corp.) was used for the calculation of expression quantities by employing the 2^-ΔΔCt^ method. To analyze the data, the ANOVA method was used. A p-value of <0.05 was considered significant.

Results: The expression levels of NOTCH1 mRNA were seen to be substantially different across groups, with a mean of 3.67±0.59, while MEC patients displayed lower overexpression at 2.48±0.53, and controls maintained baseline expression at 1.00±0.42. NOTCH1 in stage IV cancers was more upregulated (4.03±0.48) than in stage III (3.26±0.56; p<0.001).

Conclusion: The research demonstrated that NOTCH1 showed significant upregulation in oral squamous cell carcinoma and was associated with both immune suppression and unfavorable clinical outcomes. The investigation indicated that NOTCH1 stood as an effective biomarker and therapeutic target for oral cancer.

## Introduction

Oral carcinomas exist as a worldwide health threat because they typically occur in areas around the mouth, like the tongue, along with the buccal mucosa, gingiva, and floor of the mouth [1]. Oral squamous cell carcinoma (OSCC) remains the main type by constituting 90% of the cases, while mucoepidermoid carcinoma (MEC) and others occur less frequently among these types of oral carcinomas because they display various specific histopathological characteristics [2].

Oral cancer can be managed better with the help of new diagnostic and treatment methods. However, the disease remains challenging due to its slow diagnosis, frequent relapses, and poor responses to standard treatment methods. The neurogenic locus notch homolog protein 1 (NOTCH1) signalling pathway plays a major role in deciding cell fate, cell proliferation, and controlling the immune response [3]. Studies have indicated that disordered NOTCH1 expression causally links the immune system, tumour spread, and causes a negative impact on cancer therapy in carcinomas, especially oral cancers [4]. Several studies have discovered that NOTCH1 overexpression decreases the efficiency of the immune system within the tumour environment [5]. The other important signaling cascade that has been implicated in tumor survival is the PI3K/AKT signaling cascade that stimulates cell proliferation and inhibits apoptosis. The disruption of this pathway is related to resistance to conventional treatment. Of late, PI3K inhibitors are under investigation as targeted anticancer agents against a variety of cancers, including oral squamous cell carcinoma, and they may provide a synergistic strategy with NOTCH1-directed therapy.

Collaborative studies have substantially contributed to the understanding of NOTCH1 expression in different oral cancer types, since this knowledge helps in guiding how patients are treated [6]. If NOTCH1 biomarkers are accurately assessed for diverse clinical traits, including immunological responses and patterns of therapeutic reaction, there can be advances in personalized treatments for oral cancer as part of precision medicine [7,8]. Moreover, in order to clarify the exact role of NOTCH1 in the development of oral cancer, it was necessary to explore its influence on the function of the immune system.

Thus, the main objective of this study was to examine the level of NOTCH1 mRNA expression in blood and tissues of patients with OSCC and MEC, and compare them to healthy individuals. The study also aimed to examine the correlation between NOTCH1 expression and clinical staging, with the goal of determining whether NOTCH1 could serve as a prognostic biomarker and potential therapeutic target in oral cancer. By exploring these associations, the study sought to contribute to the development of more specific, immune-guided treatment strategies for oral malignancies.

## Materials and methods

A seven-month case-control research study took place between March 2023 and September 2023. The protocol received ethical clearance from the Institutional Review Board of the University of the Punjab, Lahore (#149-01-2022). The approval was in accordance with the Declaration of Helsinki. The researchers obtained a documented consent form from every participant before taking part in the study.

A total of 120 participants were included in the study, of which 40 (33.34%) were OSCC patients, 40 (33.33%) were MEC patients, and 40 (33.33%) were healthy controls. The control group was age- and gender-matched. The sample size was calculated using OpenEpi version 3.0.0 software (Atlanta, GA: Dean, Sullivan, Soe). Adult patients ranging from 18 to 75 years old with newly identified oral carcinoma, who were not on any medications, didn’t have any other health concerns, and who submitted the written informed consent, made up the study population. Exclusion criteria included patients who had previously undergone cancer treatment, had other comorbid diseases such as diabetes or autoimmune diseases, and patients who did not wish to sign the informed consent.

Researchers acquired 5 mL peripheral blood samples by venipuncture into ethylenediaminetetraacetic acid (EDTA) tubes. Tissue samples (100-200 mg) were obtained during surgery, and both samples were placed under -80°C storage using dry ice. A standardized data collection form, founded on WHO cancer registry formats, was used to record clinical data on patients, such as demographic data, tumor type, and staging.

Blood samples underwent total RNA extraction through the QIAamp Blood RNA Kit #51104 (Hilden, Germany: QIAGEN) per the manufacturer's protocol. Total RNA was isolated under RNase-free conditions by following the manufacturer's instructions. The purity and concentration of RNA were determined by a NanoDrop spectrophotometer (Waltham, MA: Thermo Fisher Scientific Inc.) (A260/A280 ratio), and only samples with ratios between 1.8 and 2.0 were considered for further analyses.

The 2^-ΔΔCt^ normalization method, which was applied to housekeeping genes, allowed proper calculation of relative NOTCH1 expression values from three replicate reactions. Even though the RT-qPCR was the major technique used to measure the gene expression, in future research, immunohistochemistry (IHC) or western blot analysis could be employed to confirm the alterations at the protein level, enabling a more significant comparison of molecular markers in the patient and control groups. All the qPCRs were performed in triplicate. There were no-template controls (NTCs) and reverse transcription minus (RT-) controls to ensure there was lack of contamination or genomic DNA. The researchers conducted statistical analyses through SPSS version 20.0 (Armonk, NY: IBM Corp.). Research participants were described through descriptive statistics (mean±SD). The study evaluated and statistically analyzed NOTCH1 expression differences in OSCC tissues, MEC specimens, and normal tissue samples through a one-way Analysis of Variance (ANOVA) test with post-hoc Tukey pairwise comparisons. The Shapiro-Wilk test was employed to test data normality, and the homogeneity of variances was verified with the Levene test prior to the ANOVA implementation. Intergroup comparison was carried out using post-hoc Tukey test. A p-value <0.05 was considered statistically significant.

## Results

The research included a total of 120 participants, composed of 80 (66.67%) oral carcinoma patients and 40 (33.33%) individuals as a control group. The oral cancer patient group included 40 (33.34%) OSCC cases and 40 (33.33%) MEC cases. Patients had a mean age of 47.6 years and a gender distribution showing 58 (72.5%) male patients versus 22 (27.5%) female patients, yielding a male-to-female ratio of 2.6:1. The control participants had a mean age of 45.3 ± 10.8 years, with a slightly unequal sex distribution: 25 males (62.5%) and 15 females (37.5%). A positive family history of cancer was reported by 57/80 (71.3%) patients, while controls did not report any family history information.

Of all patients diagnosed with MEC or OSCC, 42 (52.5%) had their cancer classified as stage III, while 38 (47.5%) had tumors designated as stage IV. The clinical course of OSCC proved aggressive, as stage IV diagnosis applied to most cases, 25 out of 40 (62.5%) cases. Stage III cancer was more common among MEC patients, with 27 out of 40 (67.5%) cases diagnosed at this stage. A breakdown of patient characteristics is presented in Table 1.

**Table 1:** Demographic and clinical characteristics of study participants. Data are presented as n (%) or mean±SD, where applicable. Descriptive statistics were used to summarize the baseline characteristics of the participants. OSCC: oral squamous cell carcinoma; MEC: mucoepidermoid carcinoma

Variables	OSCC (n=40)	MEC (n=40)	Total patients (n=80)	Controls (n=40)
Age (mean±SD)	48.1±11.4	47.2±11.0	47.6±11.2	45.3±10.8
Males, n (%)	30 (75.0%)	28 (70.0%)	58 (72.5%)	25 (62.5%)
Females, n (%)	10 (25.0%)	12 (30.0%)	22 (27.5%)	15 (37.5%)
Family history, n (%)	31 (77.5%)	26 (65.0%)	57 (71.3%)	N/A
Stage III, n (%)	15 (37.5%)	27 (67.5%)	42 (52.5%)	N/A
Stage IV, n (%)	25 (62.5%)	13 (32.5%)	38 (47.5%)	N/A

Reverse transcription quantitative polymerase chain reaction (RT-qPCR) results demonstrated that NOTCH1 mRNA showed higher levels in tumor tissues from both OSCC and MEC patients when compared to healthy tissues (p<0.05). The results of one-way ANOVA showed that NOTCH1 expression reached statistical significance between groups (p<0.001), indicating subtype-specific NOTCH1 regulation. Mean NOTCH1 expression was calculated using 2^-ΔΔCt^ in OSCC, MEC, and controls. Error bars indicate standard deviations. As seen in Table 2, expression levels of NOTCH1 mRNA were most elevated in OSCC patients, according to the results obtained from RT-qPCR analysis.

**Table 2:** NOTCH1 expression levels in blood and tumor tissues by subtype and stage. Data are presented as mean±SD. One-way ANOVA was used to calculate F values, and Tukey's post-hoc test determined p-values. A p-value of <0.05 was considered statistically significant. OSCC: oral squamous cell carcinoma; MEC: mucoepidermoid carcinoma

Groups	Sample type	Stage III (mean±SD)	Stage IV (mean±SD)	Overall mean±SD	F-value	p-Value
OSCC (n=40)	Blood	2.98±0.51	3.31±0.64	3.14±0.59	5.12	<0.001
	Tumor tissue	3.26±0.56	4.03±0.48	3.67±0.59	26.3	<0.001
MEC (n=40)	Blood	1.95±0.43	2.32±0.61	2.12±0.53	8.47	<0.001
	Tumor tissue	2.31±0.49	2.79±0.57	2.48±0.53	11.2	<0.001

The investigation results show that NOTCH1 exhibits substantial elevation during oral cancer development, particularly in cases of OSCC, and is associated with tumor progression. The data support its potential use as both a biomarker for predicting disease outcome and a therapeutic target, especially in efforts to improve oral cancer patient outcomes.

## Discussion

Research data demonstrated that substantial NOTCH1 protein overexpression occurred consistently in oral carcinomas, specifically in OSCC and MEC cases. RT-qPCR analysis revealed that NOTCH1 exhibited noticeable overexpression in OSCC patients, with expression levels reaching 3.67±0.59 compared to MEC levels at 2.48±0.53 and normal tissue levels at 1.00±0.42. Through the identification of this pattern, overexpression of NOTCH1 was shown to contribute to cancer immune evasion and tumour aggressiveness. When tumor cells activate NOTCH1, they prevent apoptosis and continue growing in a malignant manner [9]. According to this study, NOTCH1 expression was highest in OSCC tissue, followed by MEC tissues with intermediate levels and normal tissue with the lowest baseline levels. The experimental data showed that NOTCH1 regulates the immune response, which subsequently promotes oral cancer development [10].

Although RT-qPCR validated the differential expression of NOTCH1 at the transcript level, further studies using IHC or enzyme-linked immunosorbent assay (ELISA) may provide complementary information about the protein's expression in cancer and control tissues. The addition of such indicators would improve the specificity of the diagnosis by cross-confirming molecular results with phenotype expression. According to the studies, increased levels of NOTCH1 in the tumour environment alter how immune suppression occurs [11]. An overexpression of NOTCH1 in the tumour microenvironment inactivates Bcl-2-associated X protein (BAX) and Bcl-2-associated Death promoter (BAD) proteins, which help tumour cells survive and prevent immune cells from killing them [12]. The immune escape induced by NOTCH1 could be the reason behind resistance to therapy and worse outcomes in oral cancer patients [13]. It was also found in this study that stage IV tumors contained higher NOTCH1 levels than stage III tumors in OSCC. This observation, with F-value=26.3 and p<0.001, proved NOTCH1’s role in tumor progression, with mean values of 4.03±0.48 and 3.26±0.56 in stage IV and stage III oral squamous cell carcinoma, respectively. With increased NOTCH1 expression in oral cancer tumors, the efficacy of surgical treatment decreases. Since tumors with elevated NOTCH1 expression are not effectively treated by surgery alone, additional therapies such as radiotherapy, chemotherapy, and molecularly targeted agents should also be considered [14].

Clinical practitioners can use NOTCH1 measurement as both a predictive marker and a decision-support tool for designing individual treatment plans. Research studies validate the role of NOTCH1 in tumor cell proliferation and immune evasion mechanisms in head and neck cancers while demonstrating that increased expression levels are predicted at higher stages and have poor clinical outcomes [15]. Studies have shown that elevated NOTCH1 protein levels lead to unfavorable therapeutic responses and diminished survival rates among patients with OSCC [16]. Our findings are consistent with previous research results, which validate NOTCH1’s potential for clinical diagnosis and treatment applications. Although our findings prove that the higher expression of NOTCH1 is statistically significantly associated with the higher stage of cancer, there is a possibility of other mechanisms (e.g., simultaneous activation of other oncogenic signaling pathways, e.g., PI3K/AKT or EGFR) to play a role in immune evasion and tumor progression. The described patterns of overexpression should therefore be interpreted within the context of a multifactorial disease environment. In addition, despite the convincing results, the research sample only covered one geographical and ethnic group, and thus it might not be generalizable. The conclusions of this study are backed by the power of the ANOVA testing (F=26.3, p<0.001), yet confidence intervals and effect size reporting in future research would help support these relationships further.

Nonetheless, this study has certain limitations. Laboratory observations from the study cannot prove the existence of direct cause-and-effect relationships. Determining the function of NOTCH1 requires comprehensive functional research to explain the precise mechanisms through which it regulates immune responses in oral cancer. Additionally, this study is limited to transcript-level analysis of NOTCH1 expression and therefore doesn’t include protein-level validation and post-translational modification assessment of NOTCH1, which restricts the study of its functions on a tissue level. Future research must assess the therapeutic value of NOTCH1 inhibitory treatments for oral cancer through time-based clinical trials. Functional assays must be performed to get a deeper understanding of NOTCH1.

The sample was neither very large nor geographically wide, and this could impact generalizability. There is a possibility of bias in the selection of the participants, since no randomization was performed. Also, certain variables and control interventions were not noted, such as lifestyle factors or comorbidities, which might affect gene expression. Future research should address these limitations through more robust, multicenter study designs and broader monitoring of relevant variables.

## Conclusions

The research indicates that NOTCH1 is a critical biomarker in oral carcinoma pathophysiology. Studies have identified that cancer stages with immune suppression are strongly associated with elevated NOTCH1 expression levels. The data indicate that NOTCH1 functions both as a strong marker for forecasting patient outcomes and as a targetable therapeutic solution.

Clinical strategies for personalized care in oral carcinoma patients can be advanced through the combined execution of NOTCH1-targeted intervention techniques with precision-based surgeries. More studies need to analyze the effectiveness of NOTCH1 inhibitors clinically, as well as to develop immune-modulating treatments, to enhance patient outcome results.
